# Supplementary material for: Brain-derived neurotrophic factor supports pericyte and vascular homeostasis in the aging brain
Source: Acta Neuropathol Commun. 2025 Dec 1;13:246. doi: 10.1186/s40478-025-02181-y (PMC12667088; doi:10.1186/s40478-025-02181-y)
Supplement: Supplementary file 1 — Supplementary Material 1. [file 40478_2025_2181_MOESM1_ESM.pdf]

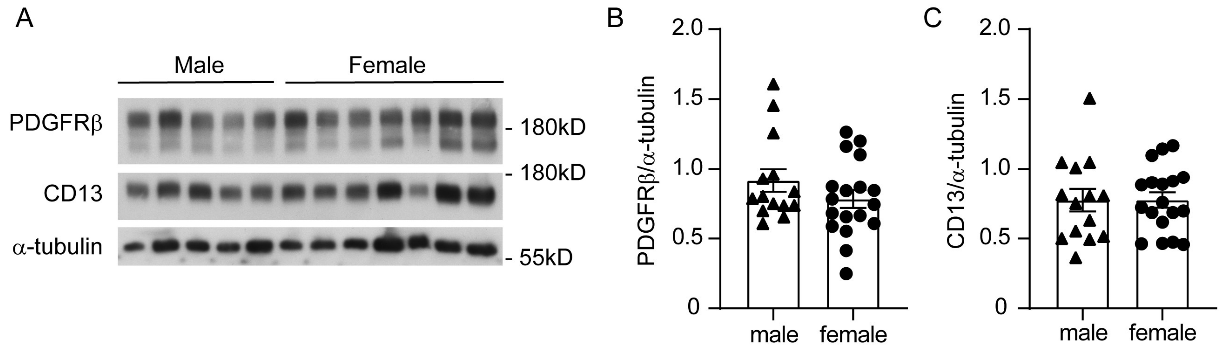

Supplementary Fig. 1, Sex has no impact on the expression of PDGFRβ and CD13 in cerebral microvessels.

(A) Cerebral microvessels were isolated from 9-month-old male and female littermate mice and homogenized for Western blot analysis of pericyte markers PDGFRβ and CD13, and α-tubulin as an internal control.

(B and C) Male and female mice do not differ in terms of PDGFRβ and CD13 protein levels (B and C; *t*-test, n = 14 and 18 for male and female mice, respectively).

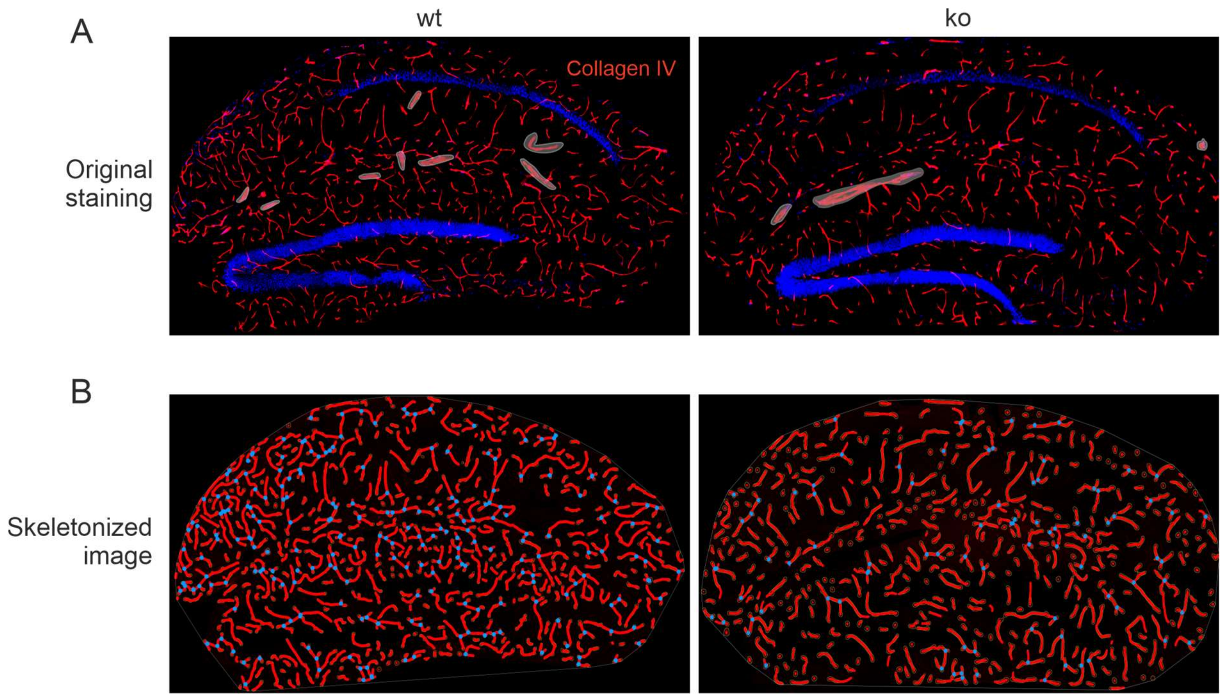

Supplementary Fig. 2, Quantification of microvessels in the brain. Brain tissue from 10-month-old C57BL6 mice with (ko) and without (wt) knockout of *Bdnf* gene in astrocytes over a period of 3 months was embedded in paraffin and cut into 30-µm sections.

(A) The brain sections were then stained for collagen type IV (in red) and imaged with Microlucida (MBF Bioscience). Blood vessels larger than 6 µm (in shadow) were excluded for further analysis.

(B) The virtual section was cropped to delineate the region of interest. The color was split and the image in the red channel was thresholded, despeckled and skeletonized. The skeleton representation of vasculature is shown in red, and branching points of the blood vessels are shown in blue. The length and branching points of blood vessels were automatically quantified using free software, AngioTool (http://angiotool.nci.nih.gov).

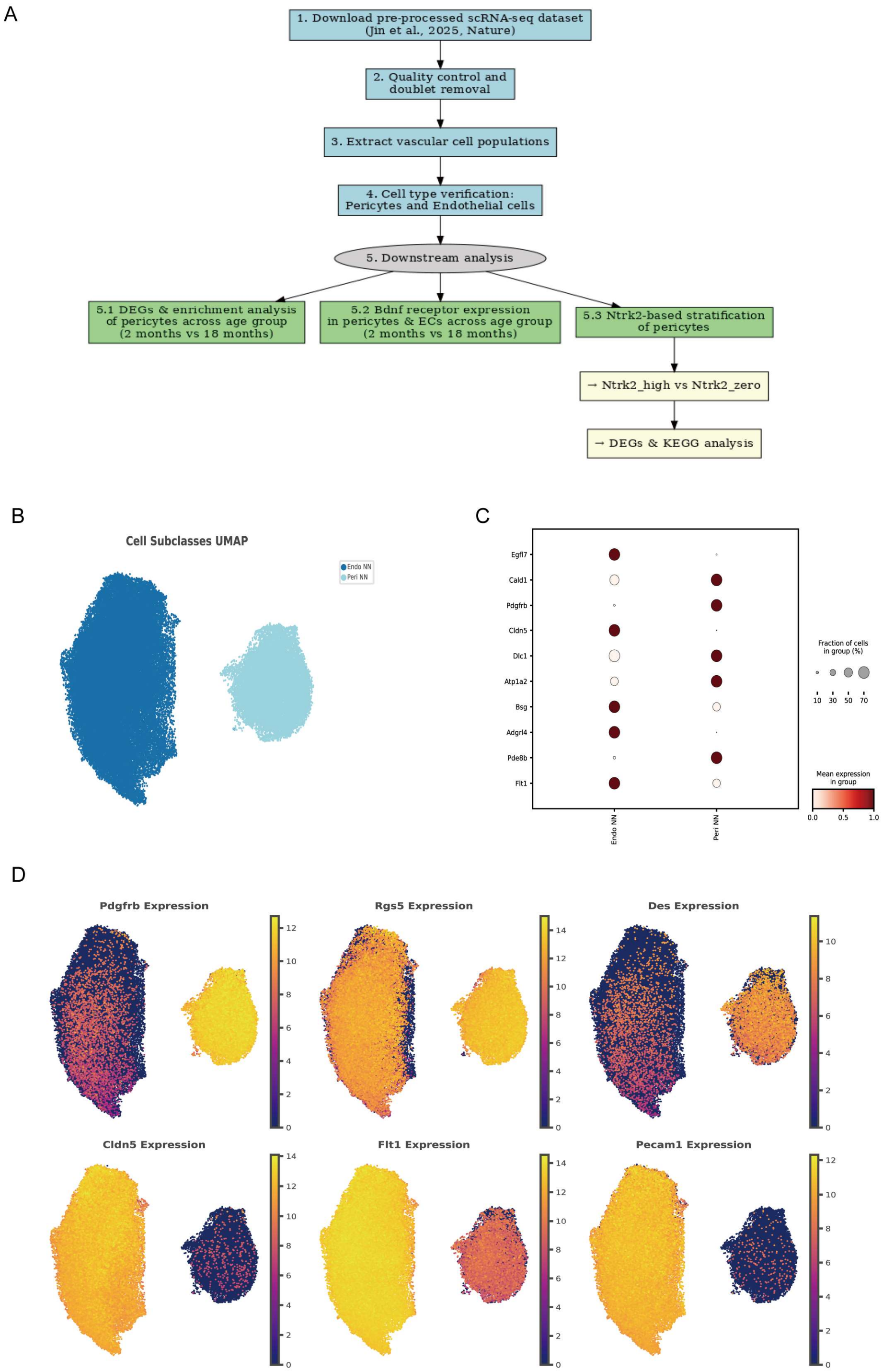

Supplementary Fig. 3, Singe-cell sequencing analysis of pericytes. (A) Workflow of scRNA-seq data analysis. Overview of the analysis pipeline including extraction of vascular cells, identification of pericytes and endothelial cells, age group comparison (2 months vs. 18 months), assessment of BDNF receptor expression, and *Ntrk2*-based stratification with downstream enrichment analysis. **(B)** UMAP visualization of extracted endothelial cells and pericytes, showing clear separation of these two vascular cell types in low-dimensional space. **(C)** Dot plot showing the top five marker genes for each vascular cell type. Dot size represents the percentage of cells expressing the gene, and color intensity reflects the average expression level. **(D)** UMAP feature plots displaying three canonical pericyte markers and three canonical endothelial markers. Yellow indicates high expression (high enrichment), and purple indicates low expression.

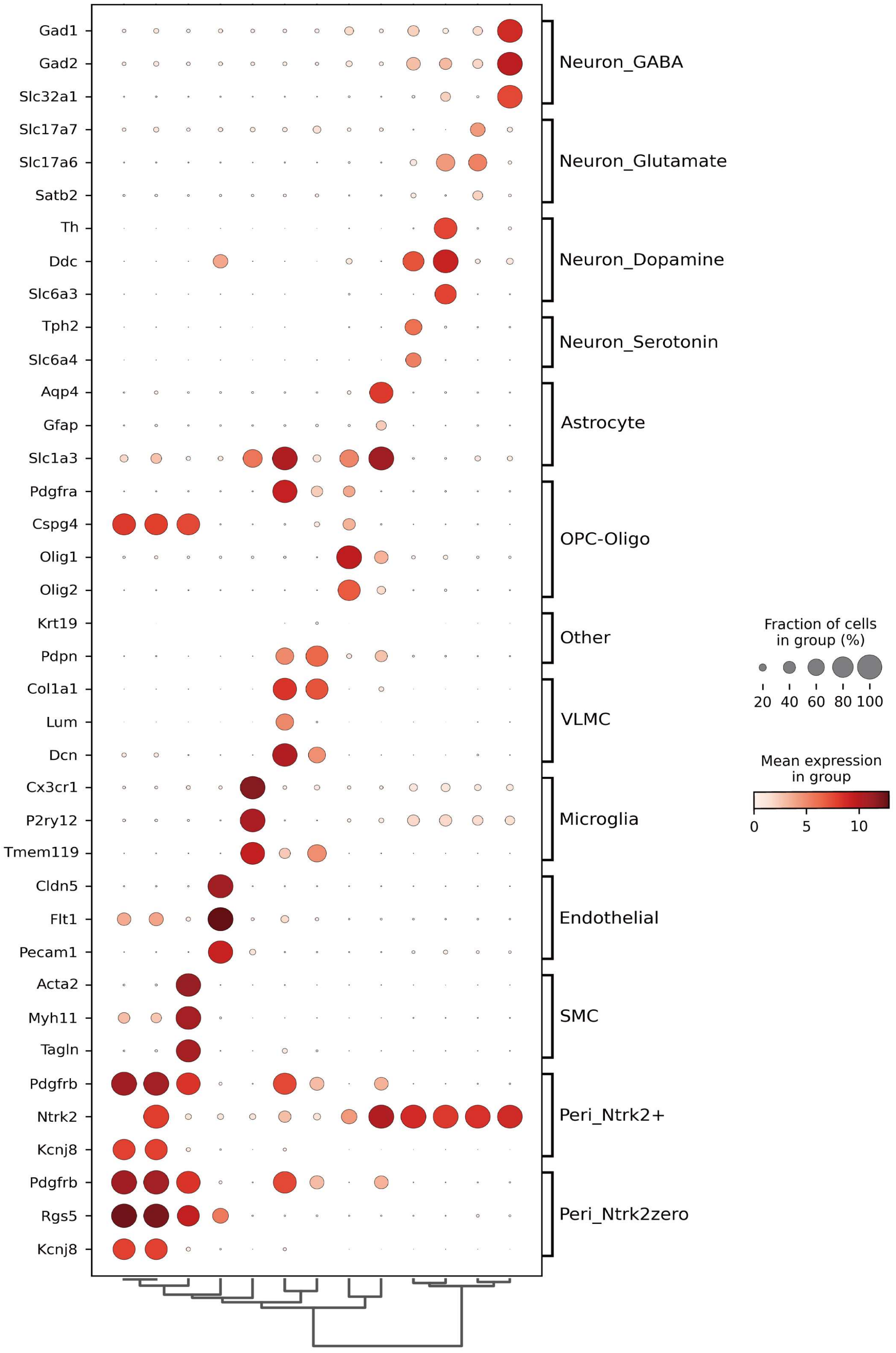

Supplementary Fig. 4, Dot plot showing the normalized average expression of canonical marker genes across brain cell types involved in cell–cell communication. The size of each dot indicates the proportion of cells within each cell type that express the corresponding gene, while the color represents the average expression level. Marker genes were selected based on well-established cell type–specific expression patterns in the mouse brain. This figure validates the plausibility of the cell identity annotations.

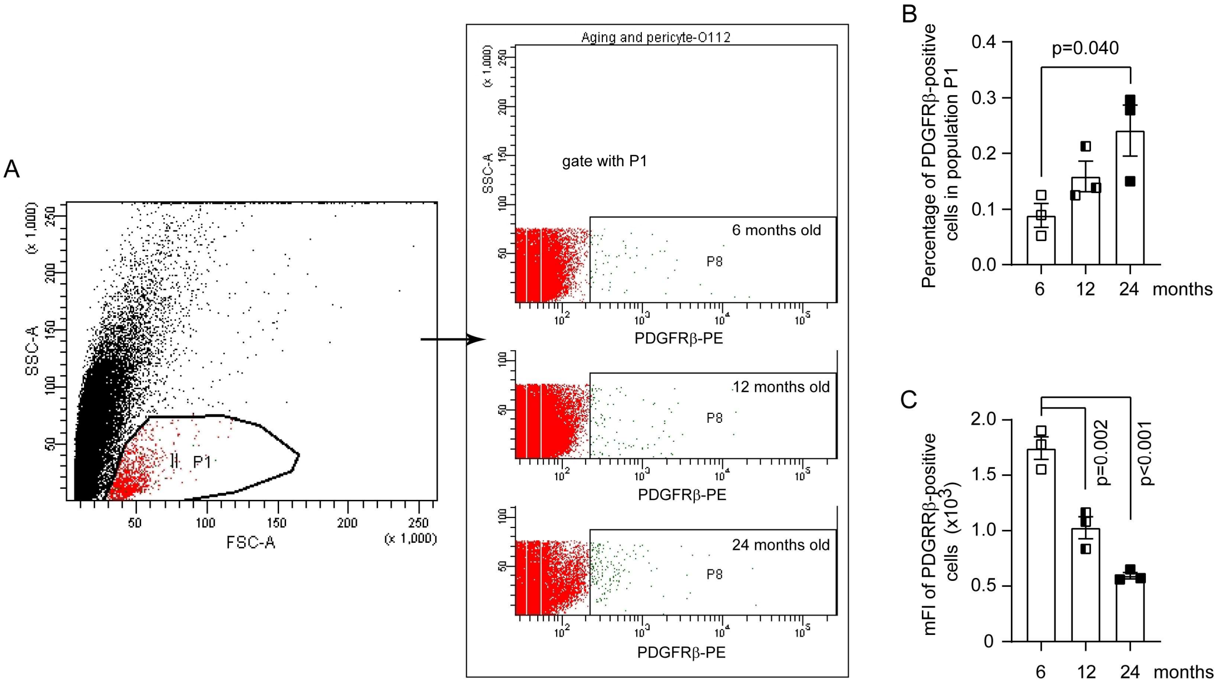

Supplementary Fig. 5, Flow cytometric analysis of pericytes in aging brains. Brain tissue (hippocampus and cortex) from 6-, 12- and 24-month-old C57BL6 mice was homogenized and digested to prepare single-cell suspensions using Neural Tissue Dissociation Kit (papain-based) (Miltenyi Biotec GmbH, Bergisch Gladbach, Germany). After blocking with 50 µg/ml CD16/CD32 antibody (clone 2.4G2; BioXCell, Lebanon, USA), brain cells were stained with PE-conjugated rat monoclonal antibody against mouse PDGFRβ (clone APB5; Miltenyi Biotec GmbH). Thereafter, the percentage and mean fluorescence intensity (mFI) were detected by BD FACSCanto™ II flow cytometry (BD Biosciences, Heidelberg, Germany).

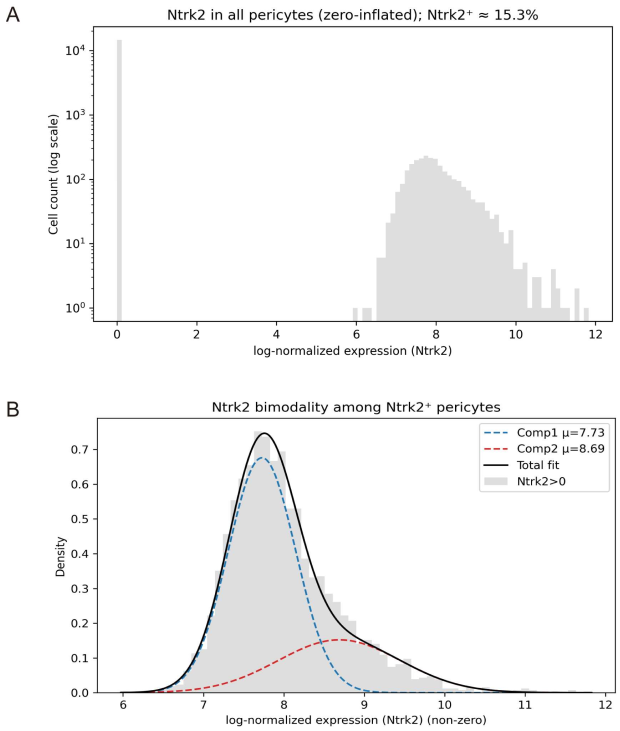

Supplementary Fig. 6, Bimodal distribution of Ntrk2 - expressing pericytes.
(A) Global distribution of log-normalized Ntrk2 expression in pericytes. The histogram shows all cells; the overlaid curve is the kernel density estimate (KDE). Zero counts comprise 84.7%, indicating pronounced zero-inflation. Hartigan's dip test was significant on the full distribution (dip = 0.053, $p < 1\times10^{-16}$), consistent with non-unimodality driven by zeros; by contrast, the test on the non-zero subset was non-significant (dip = 0.003, p = 1.000), supporting a near-unimodal positive component.
(B) Two-component Gaussian mixture (2-GMM) fitted to non-zero Ntrk2 expression. Dashed curves denote component densities; the black curve is the total density. Vertical lines indicate component means ($\mu_1 = 7.728$, $\mu_2 = 8.689$), with separation $|\Delta\mu| = 0.961$. The purple dashed line marks the operational cutoff (midpoint, 8.2089) used to define High vs Low among positives. Bayesian information criterion (BIC) favored K = 2 over K = 1 or 3 on the non-zero data. Group proportions: High 4.71%, Low 95.29%. Hartigan's dip test on the non-zero subset was non-significant (dip = 0.003, p = 1.000), consistent with a near-unimodal positive component.

Methods:
We evaluated whether Ntrk2 expression in the pericyte subset (peri_clean) is bimodal and derived an operational stratification. Log-normalized expression values were extracted from the AnnData object (sparse matrices were densified) to obtain the full expression vector (expr). To visualize the overall distribution and zero inflation, we plotted a histogram with 60 bins overlaid with a KDE (Seaborn histplot, bins=60) and reported the fraction of zero values. We then performed Hartigan's dip test (two-sided, α=0.05) at two levels: (i) all cells (expr) and (ii) the non-zero subset (expr > 0). Dip(all) captures departure from unimodality at the population level (often driven by zeros), whereas dip(non-zero) adjudicates the modality (uni- vs. bi-modal) within the positive-expression component. The dip test was run via the Python diptest package (skipped with a note if unavailable).

To characterize the shape of the non-zero distribution, we fitted Gaussian Mixture Models (GMMs; scikit-learn) to the non-zero subset and compared BIC across K = 1, 2, 3, selecting the model with the lowest BIC. When K = 2 was preferred, we reported component means and weights and their separation **|Δμ|-|Δμ|**. This GMM/BIC analysis describes skewness/heavy tails and does not itself constitute statistical evidence of bimodality, which relies on dip(non-zero). For downstream contrasts and visualization, when a 2-GMM was available we used the midpoint between the two component means as an operational threshold to label all pericytes as High/Low (Ntrk2_bimodal_group), and we reported group proportions. Analyses used random state=42; figures were generated with matplotlib/seaborn, and the BIC table plus a compact JSON summary were saved to ensure reproducibility.
