# Supplementary figures and images for: Brain-derived neurotrophic factor supports pericyte and vascular homeostasis in the aging brain
Source: Acta Neuropathol Commun. 2025 Dec 1;13:246. doi: 10.1186/s40478-025-02181-y (PMC12667088; doi:10.1186/s40478-025-02181-y)

Fig. 2, A

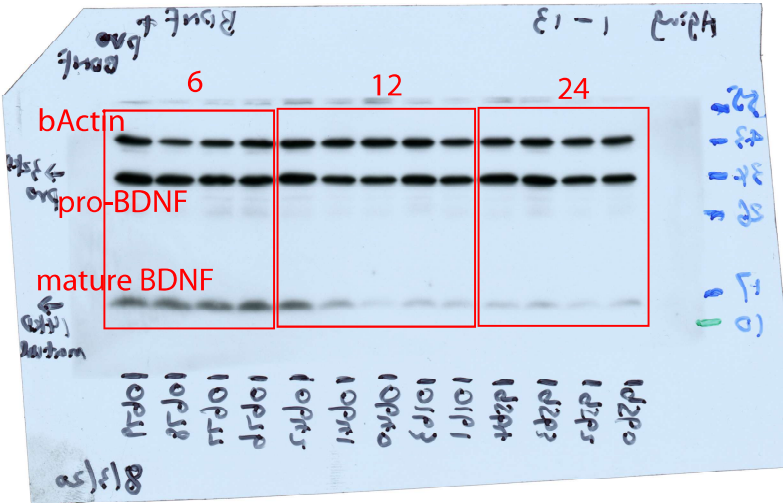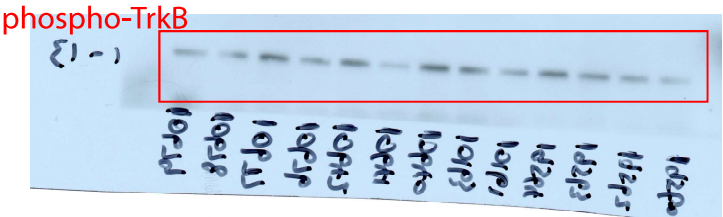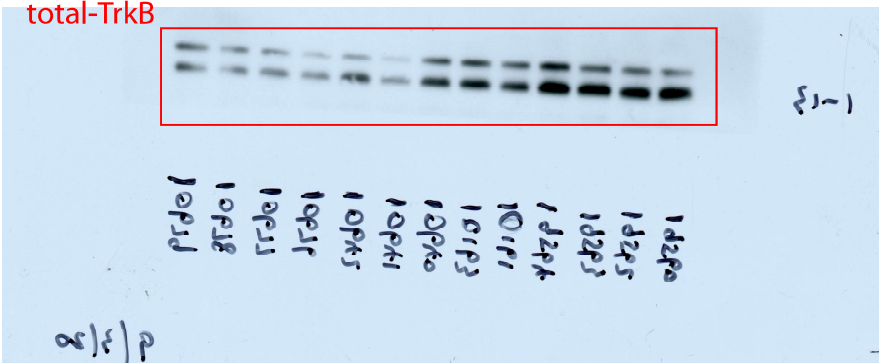

Fig. 3, F

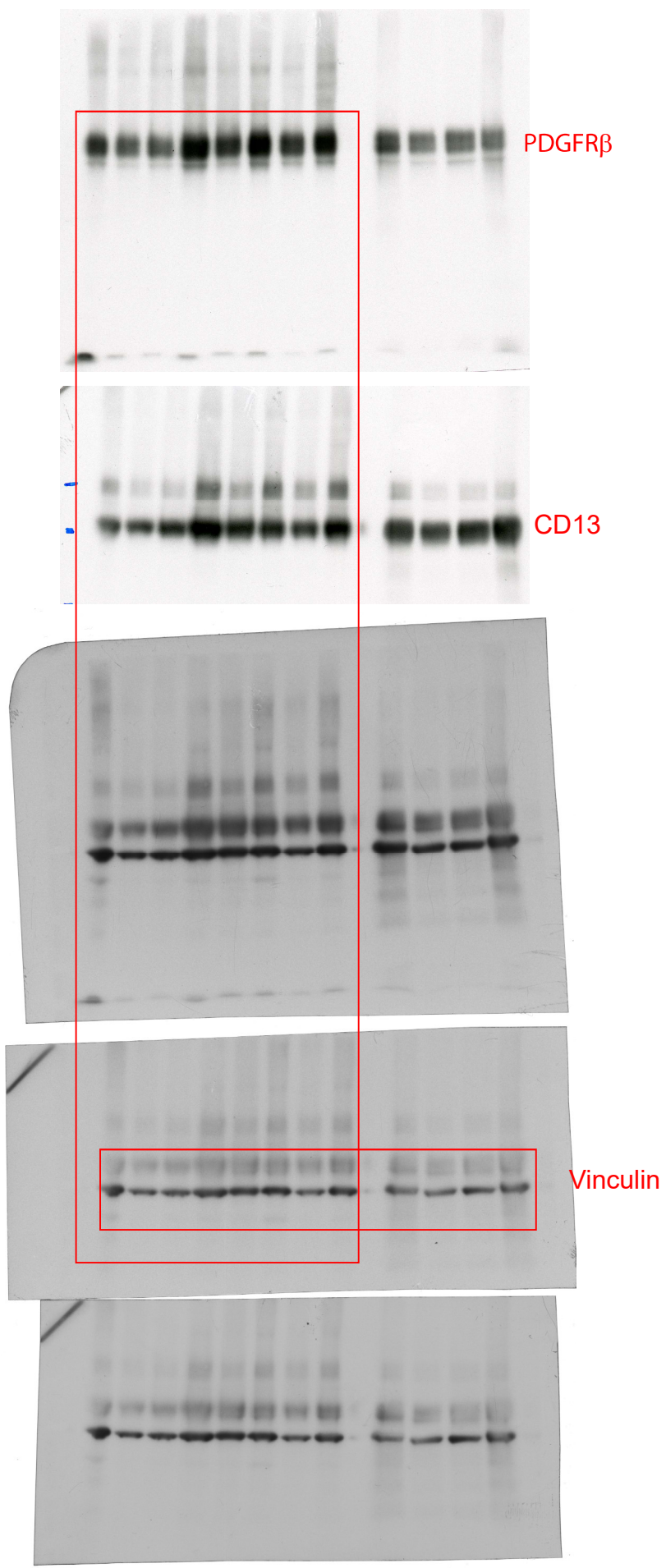

Fig. 3, I

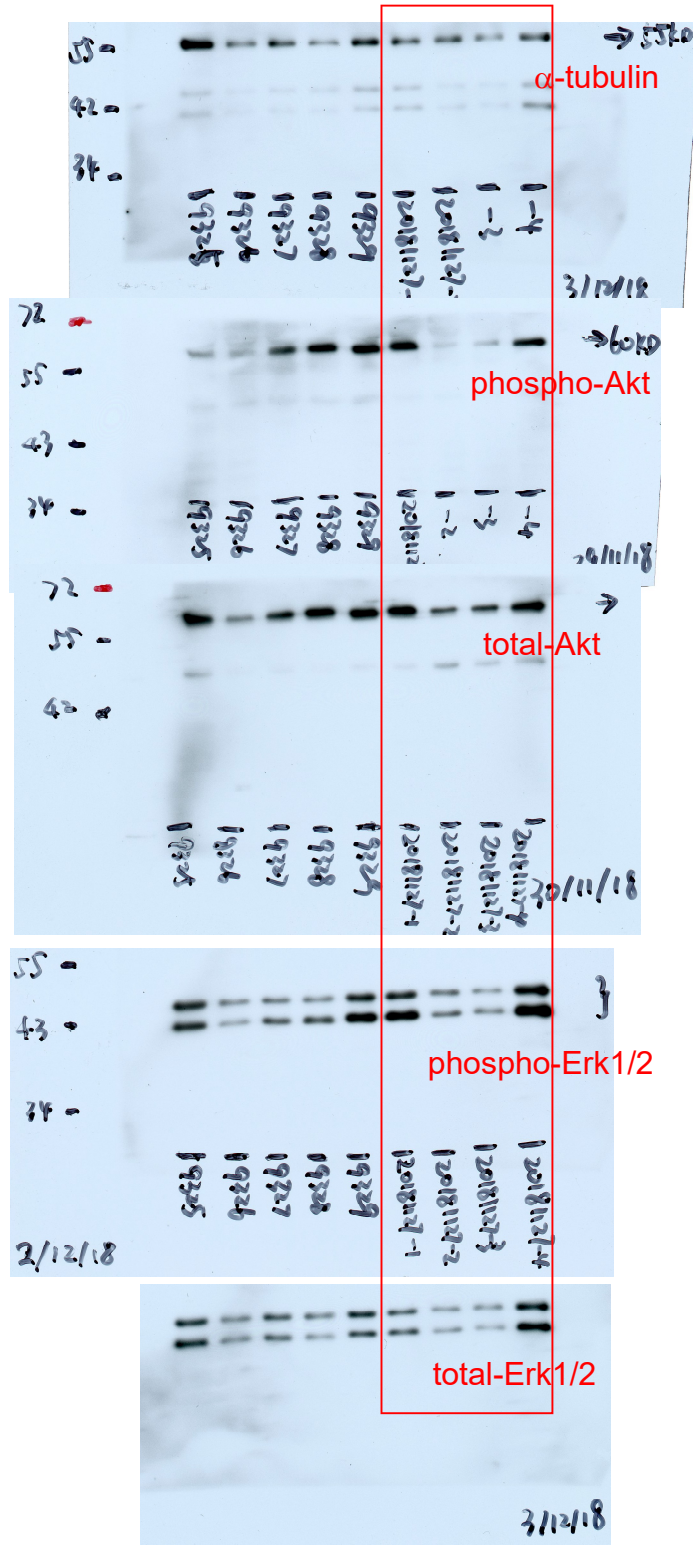

Fig.4, G

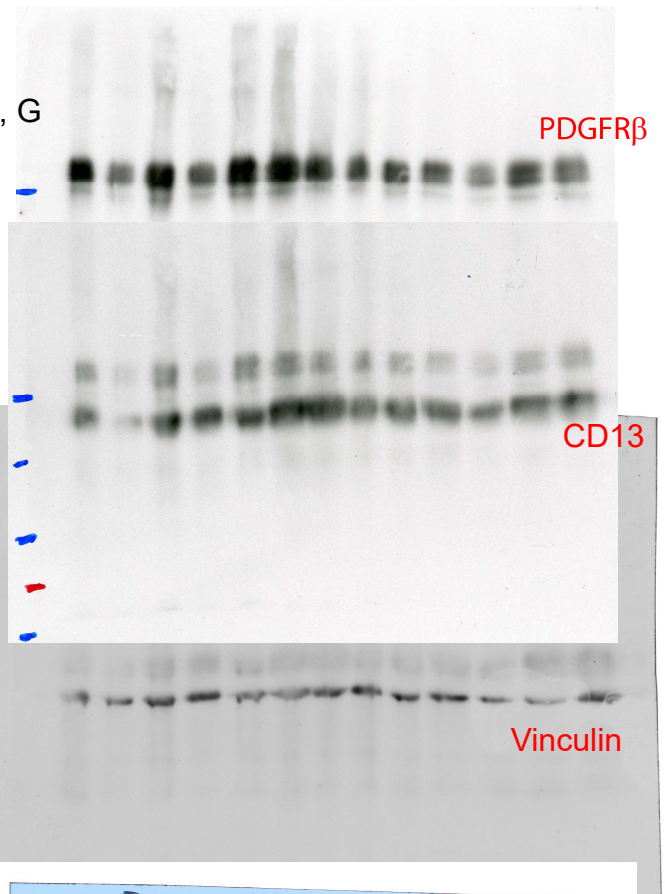

Fig.4, A

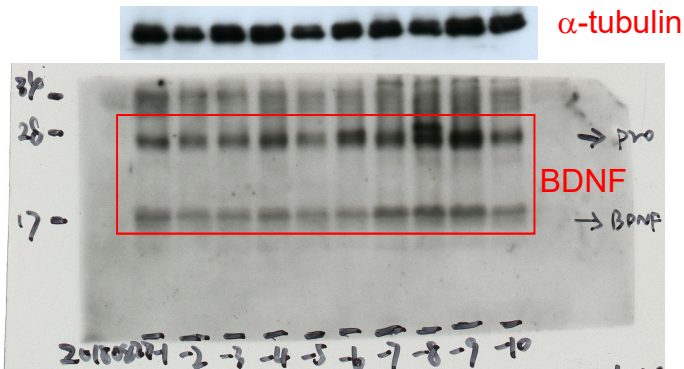

Fig. 4, J

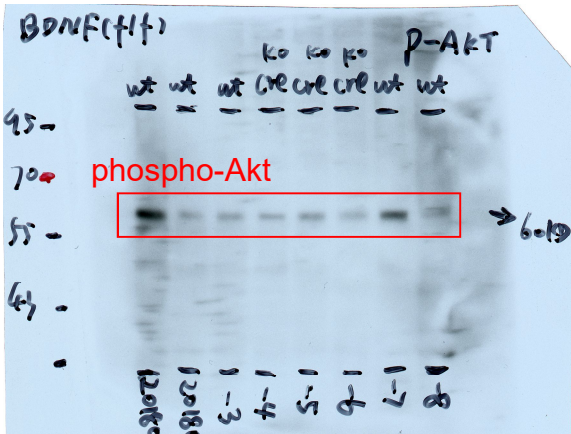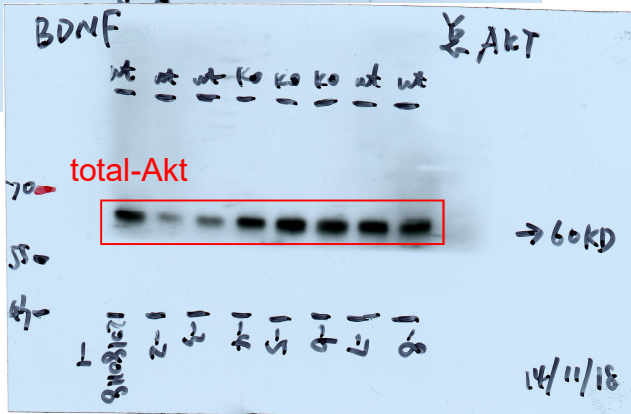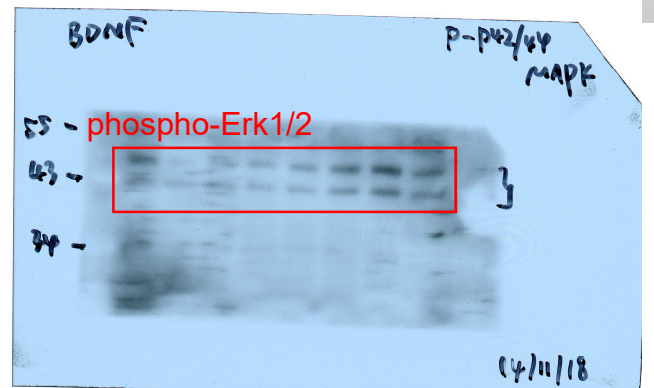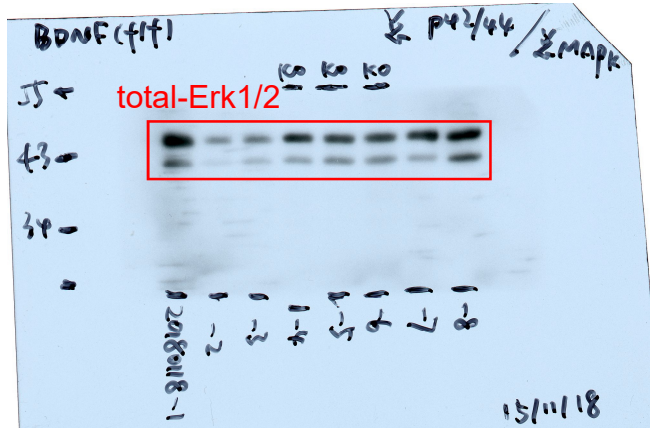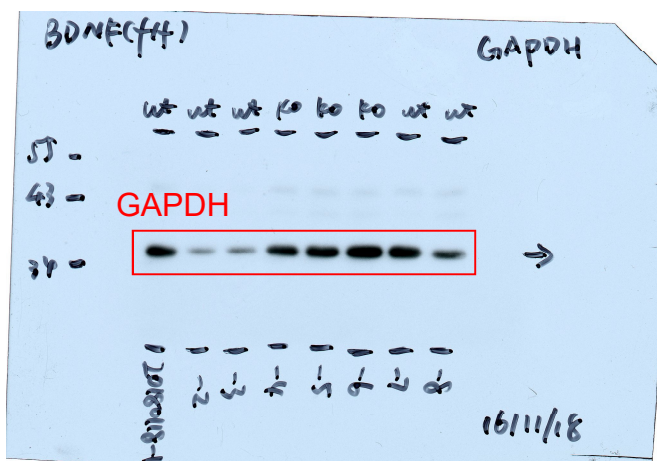

Fig. 6, C

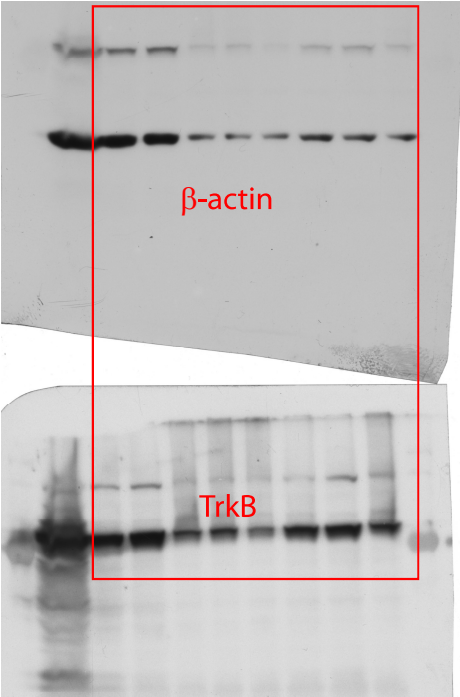

Fig. 6, D

Fig. 6, H

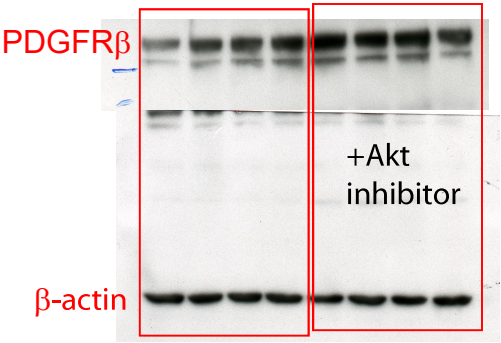

Fig. 6, D

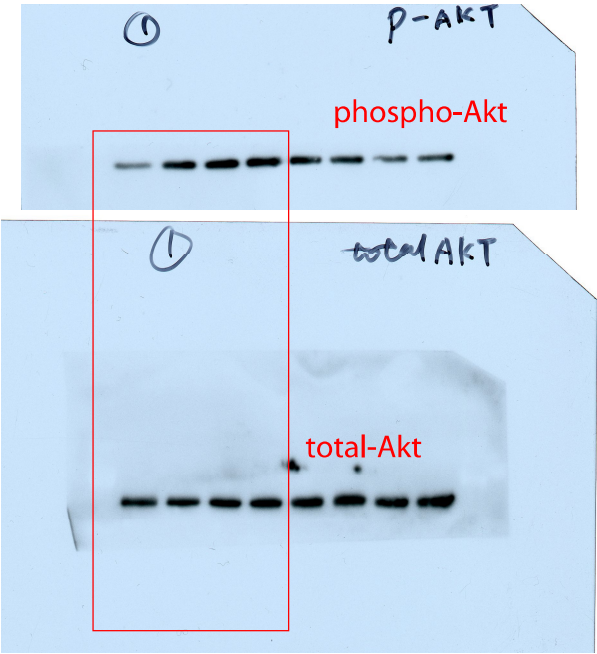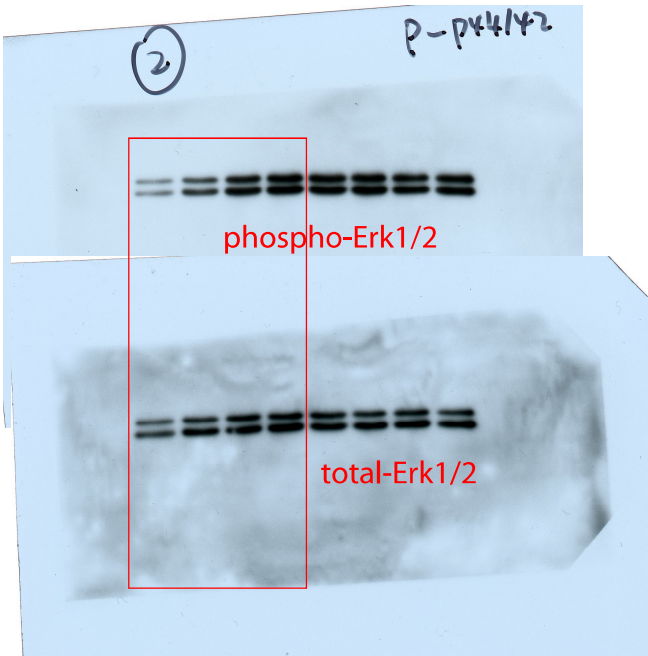

Supplementary Figure 1

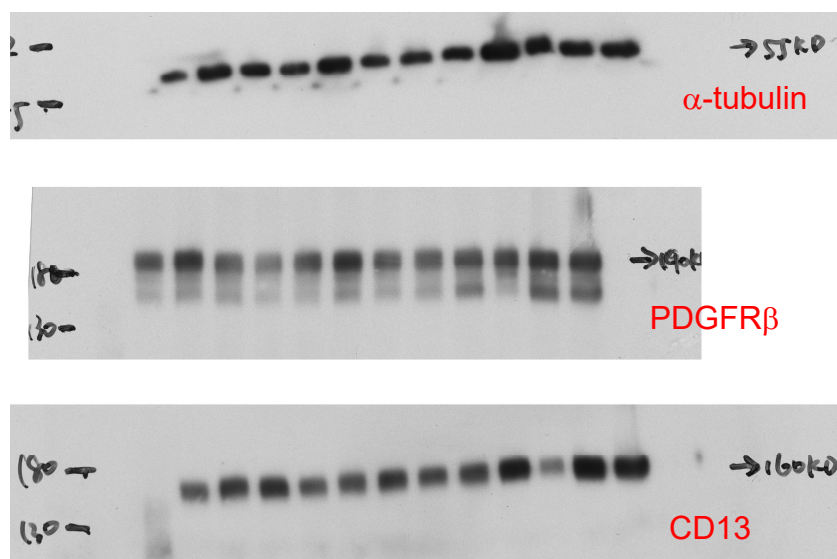

Supplement: Supplementary file 3 — Supplementary Material 3. [file 40478_2025_2181_MOESM3_ESM.pdf]
